# Supplementary figures and images for: Loss of N1-methylation of G37 in tRNA induces ribosome stalling and reprograms gene expression
Source: eLife. 2021 Aug 12;10:e70619. doi: 10.7554/eLife.70619 (PMC8384417; doi:10.7554/eLife.70619)

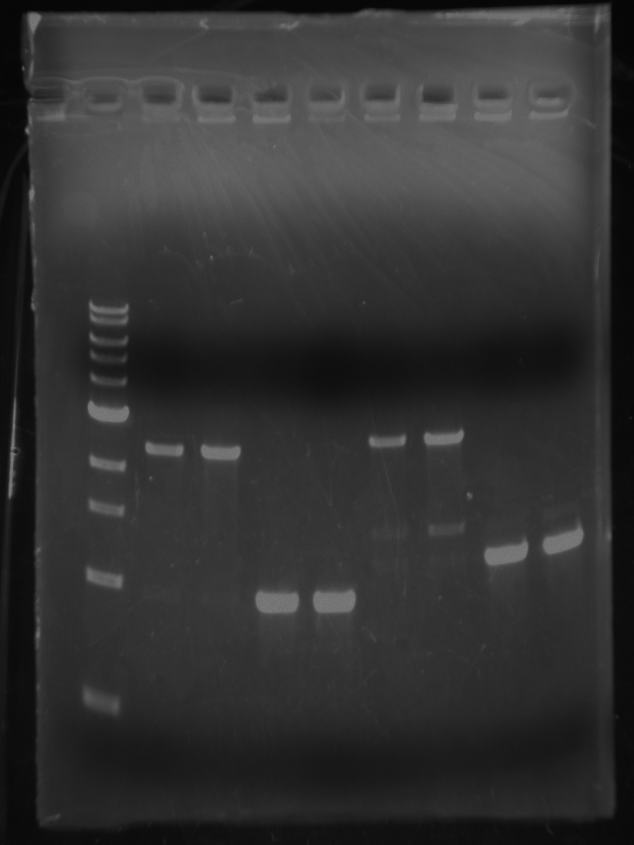

Supplement: Source data 1. [file elife-70619-data1.zip › Figure 1-figure supplement 1-source data 1 raw.tif]

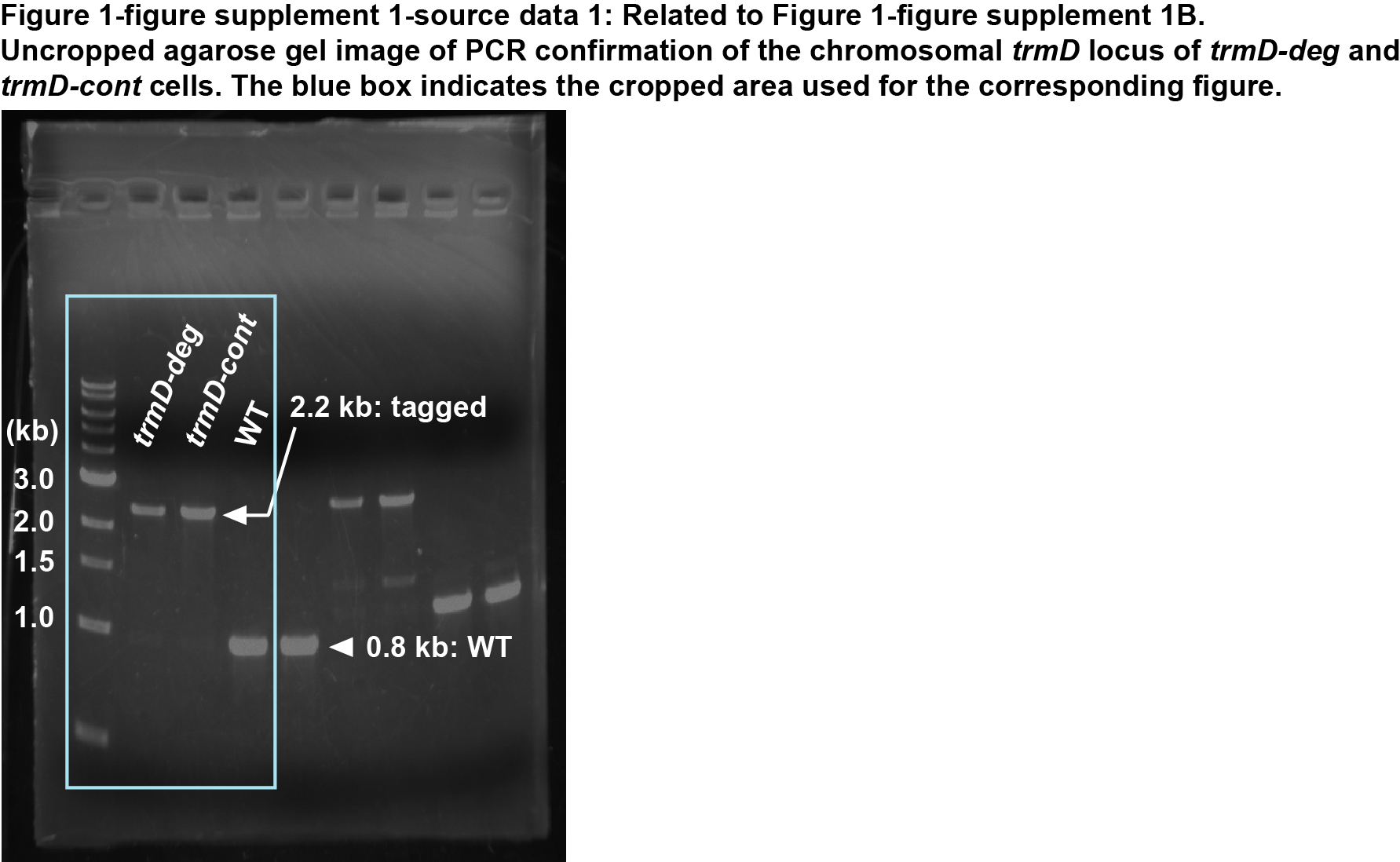

Supplement: Source data 1. [file elife-70619-data1.zip › Figure 1-figure supplement 1-source data 1.jpg]

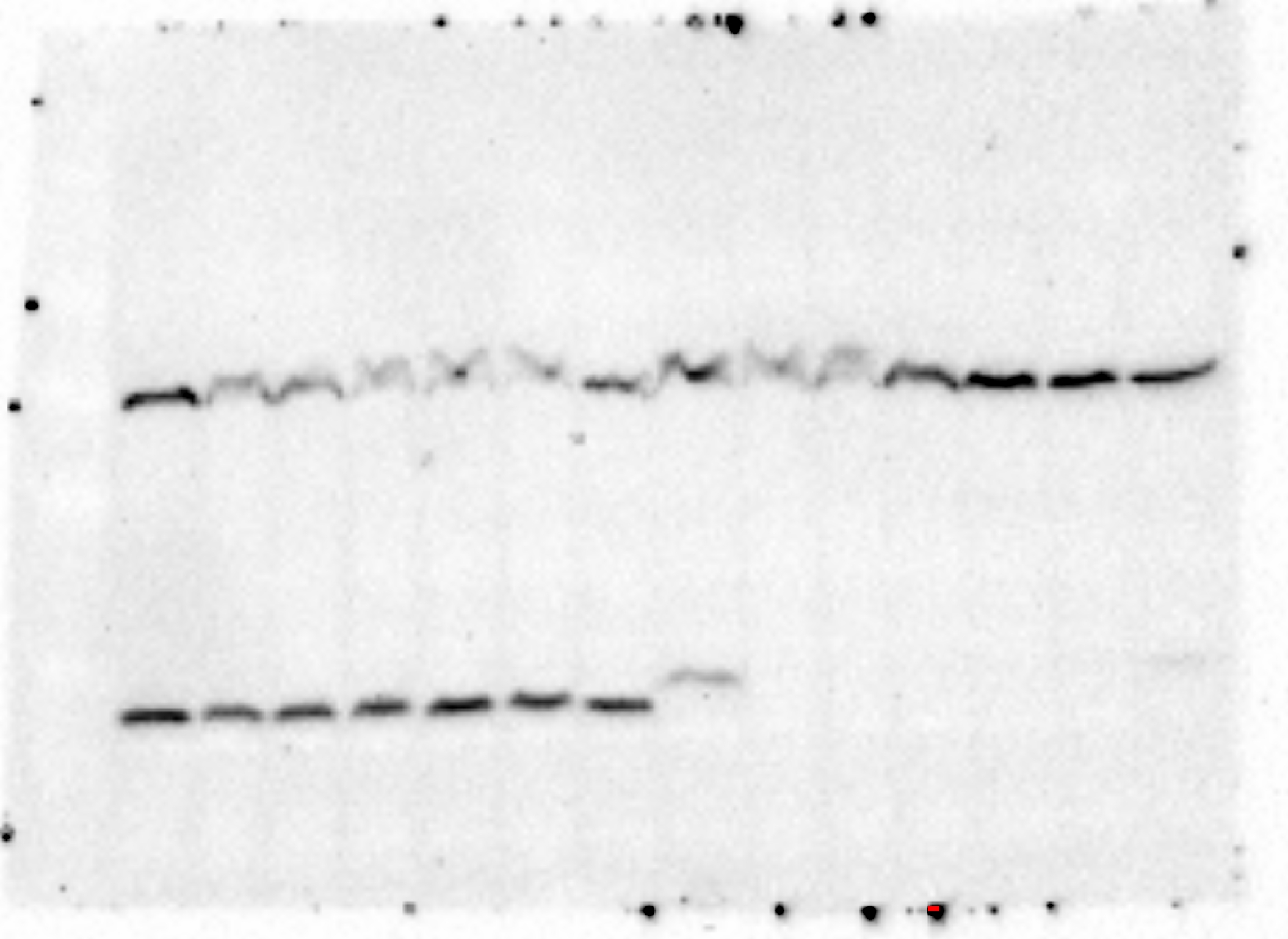

Supplement: Source data 1. [file elife-70619-data1.zip › Figure 1-figure supplement 1-source data 2 raw.jpg]

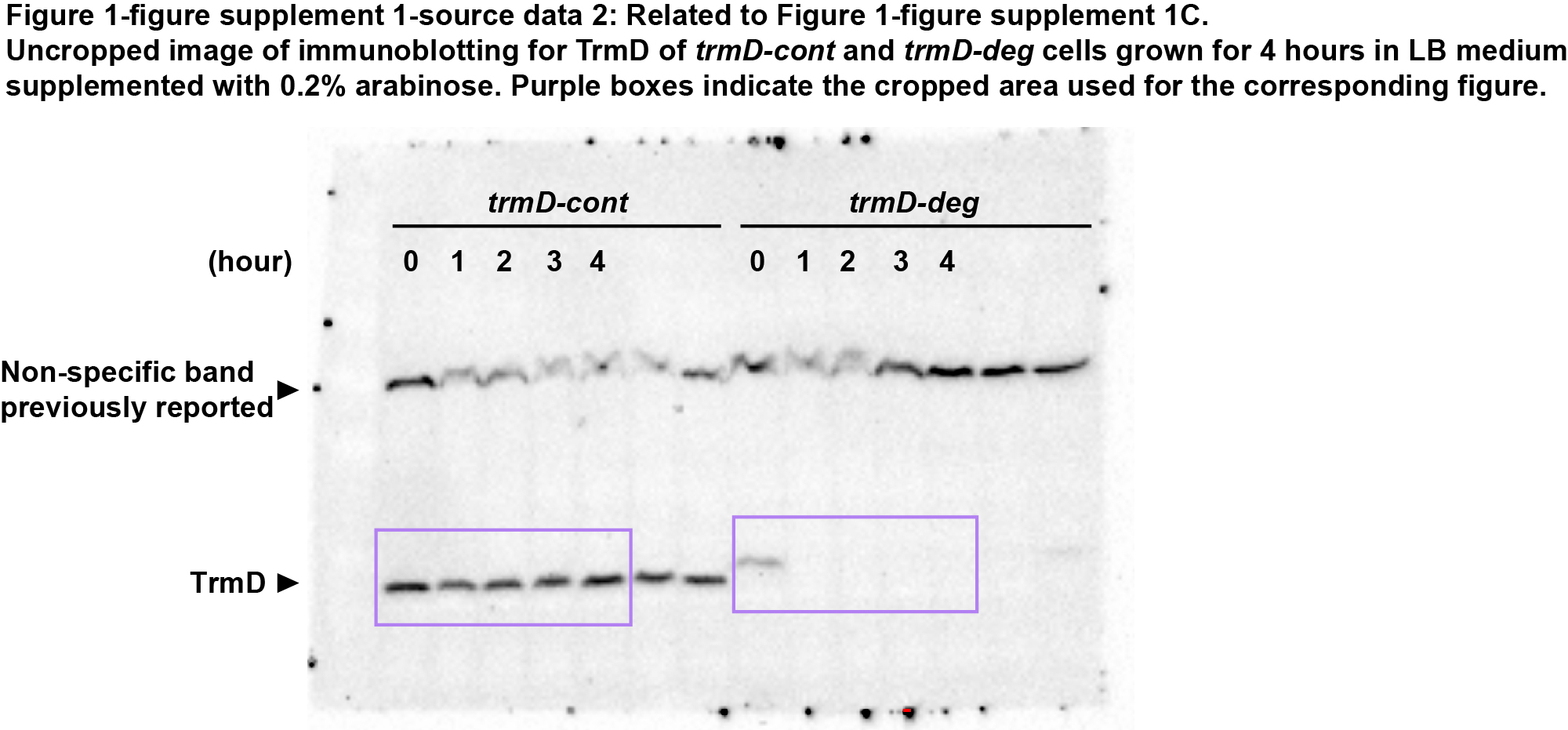

Supplement: Source data 1. [file elife-70619-data1.zip › Figure 1-figure supplement 1-source data 2.jpg]

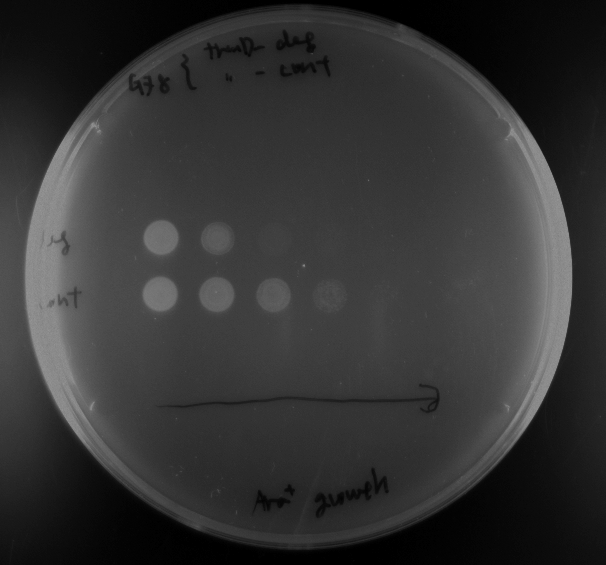

Supplement: Source data 1. [file elife-70619-data1.zip › Figure 1-source data 1 raw.tif]

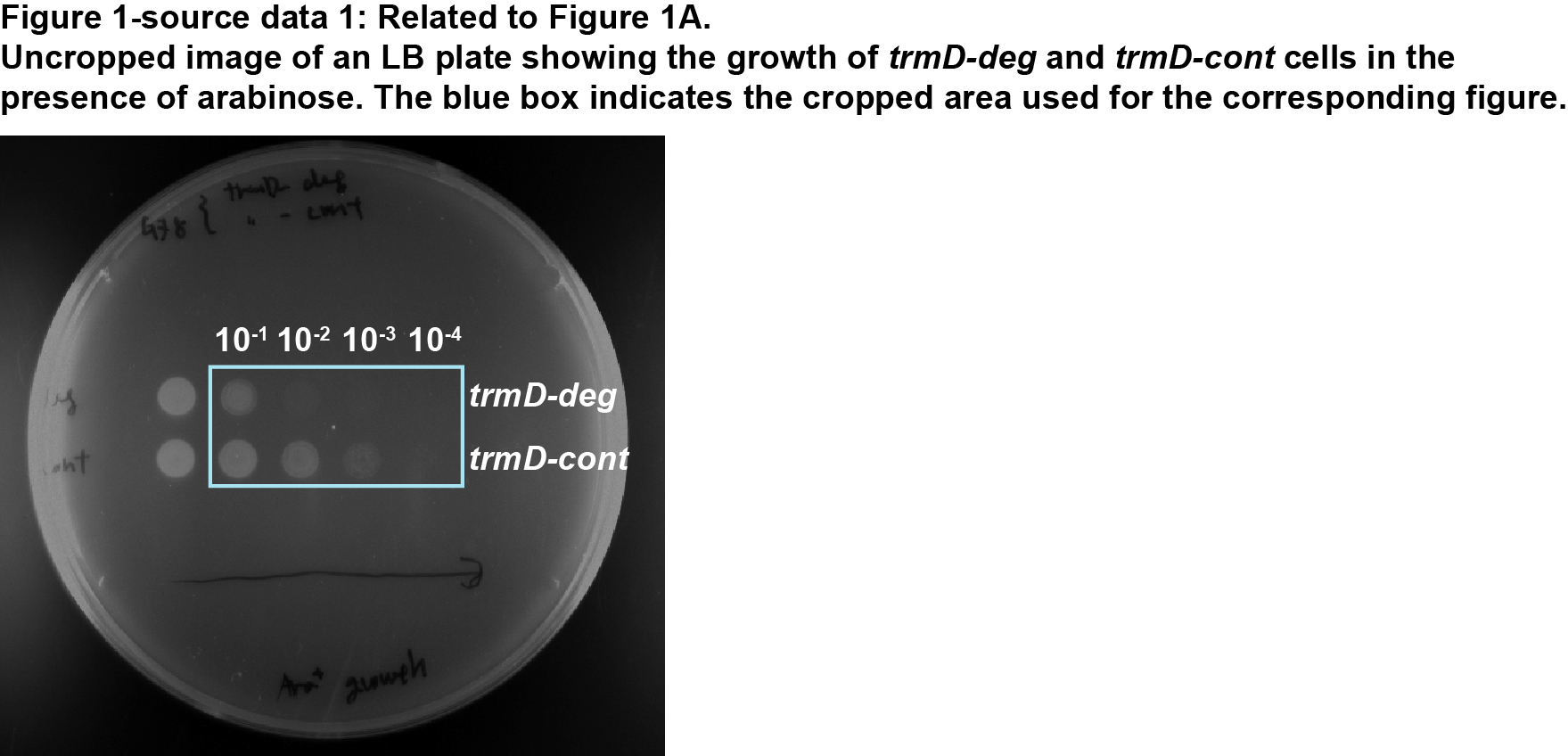

Supplement: Source data 1. [file elife-70619-data1.zip › Figure 1-source data 1.jpg]

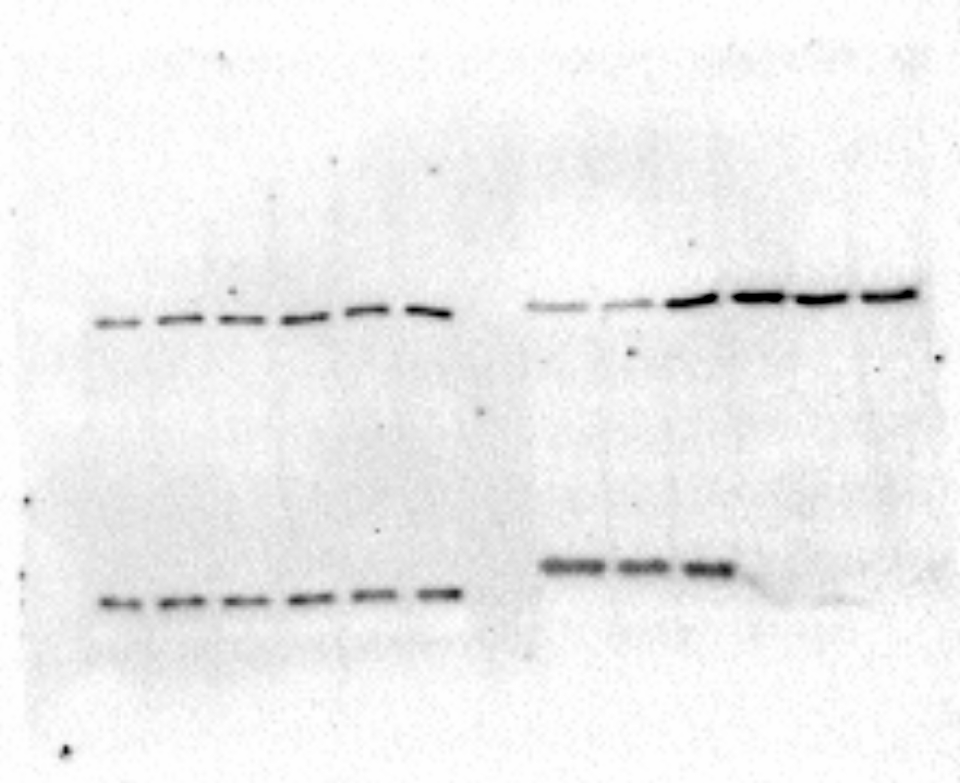

Supplement: Source data 1. [file elife-70619-data1.zip › Figure 1-source data 2 raw.jpg]

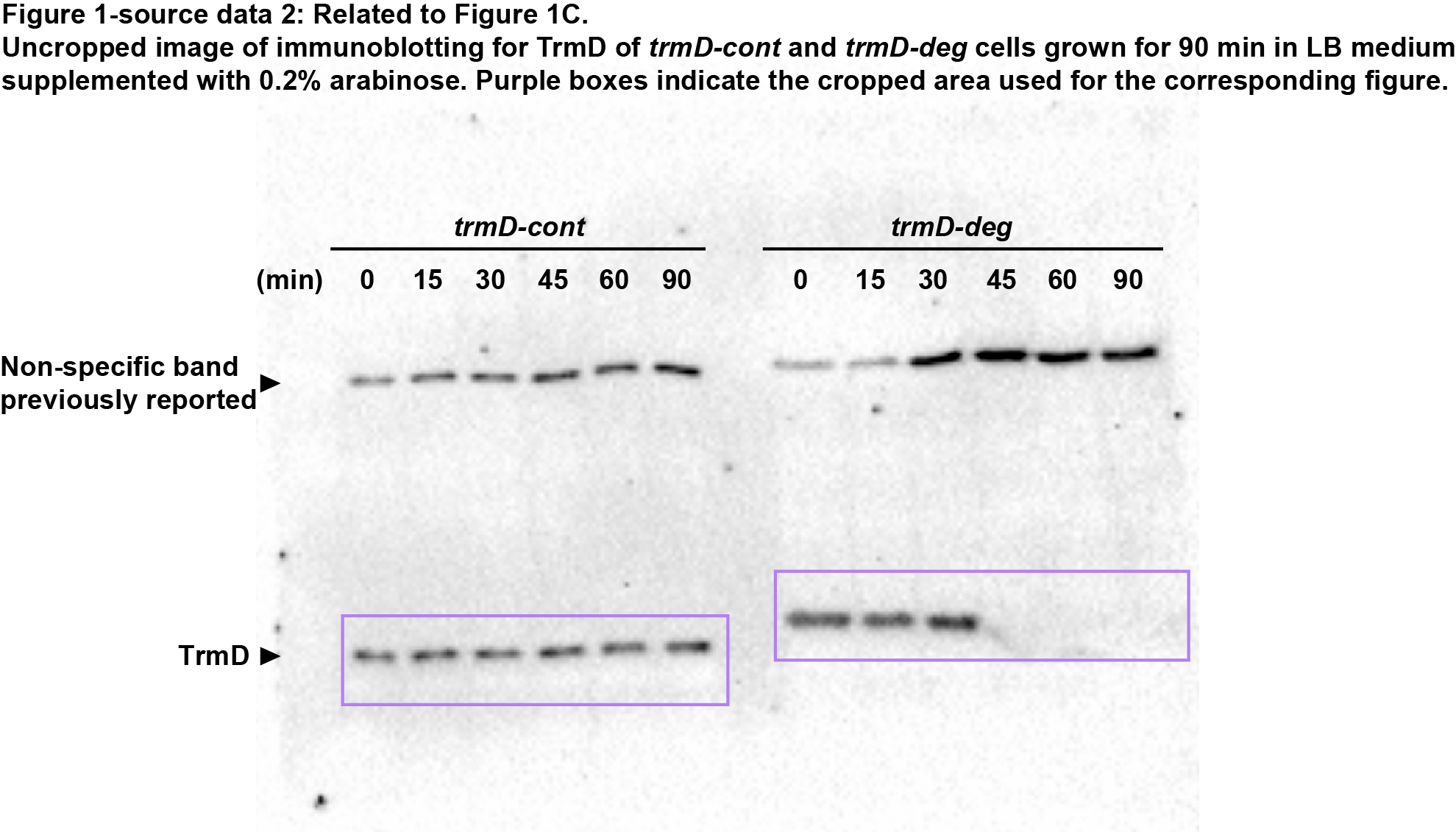

Supplement: Source data 1. [file elife-70619-data1.zip › Figure 1-source data 2.jpg]

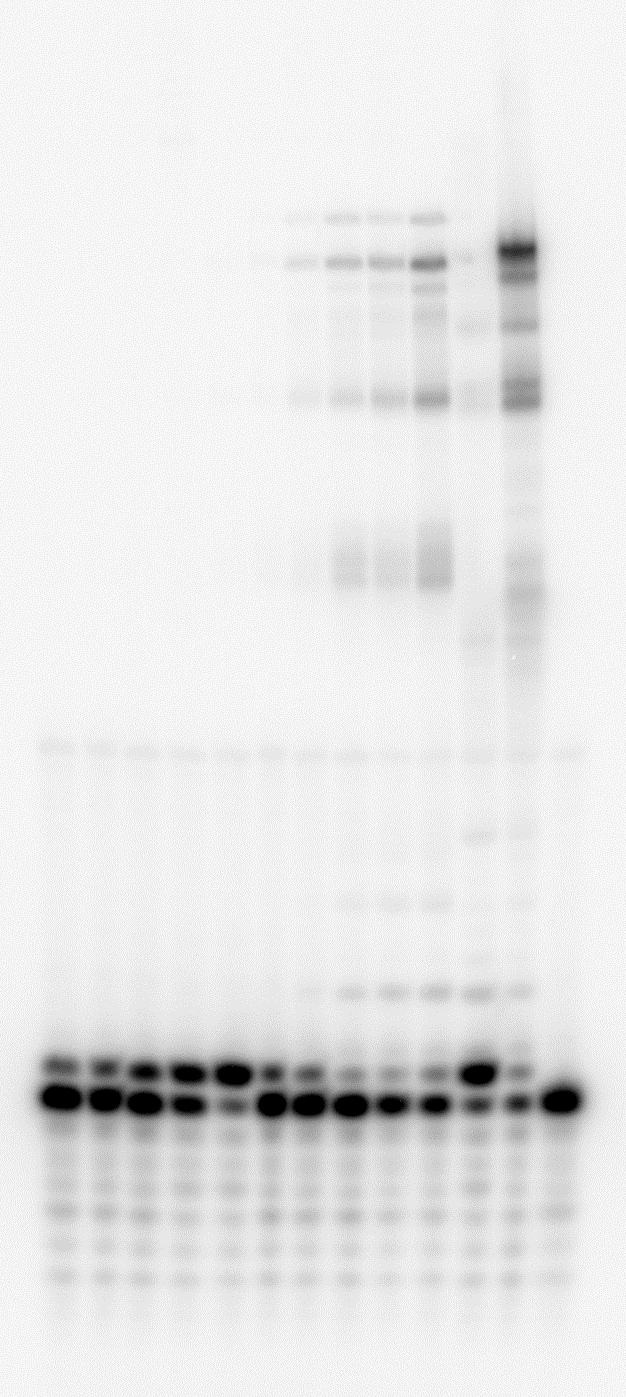

Supplement: Source data 1. [file elife-70619-data1.zip › Figure 1-source data 3 raw.tif]

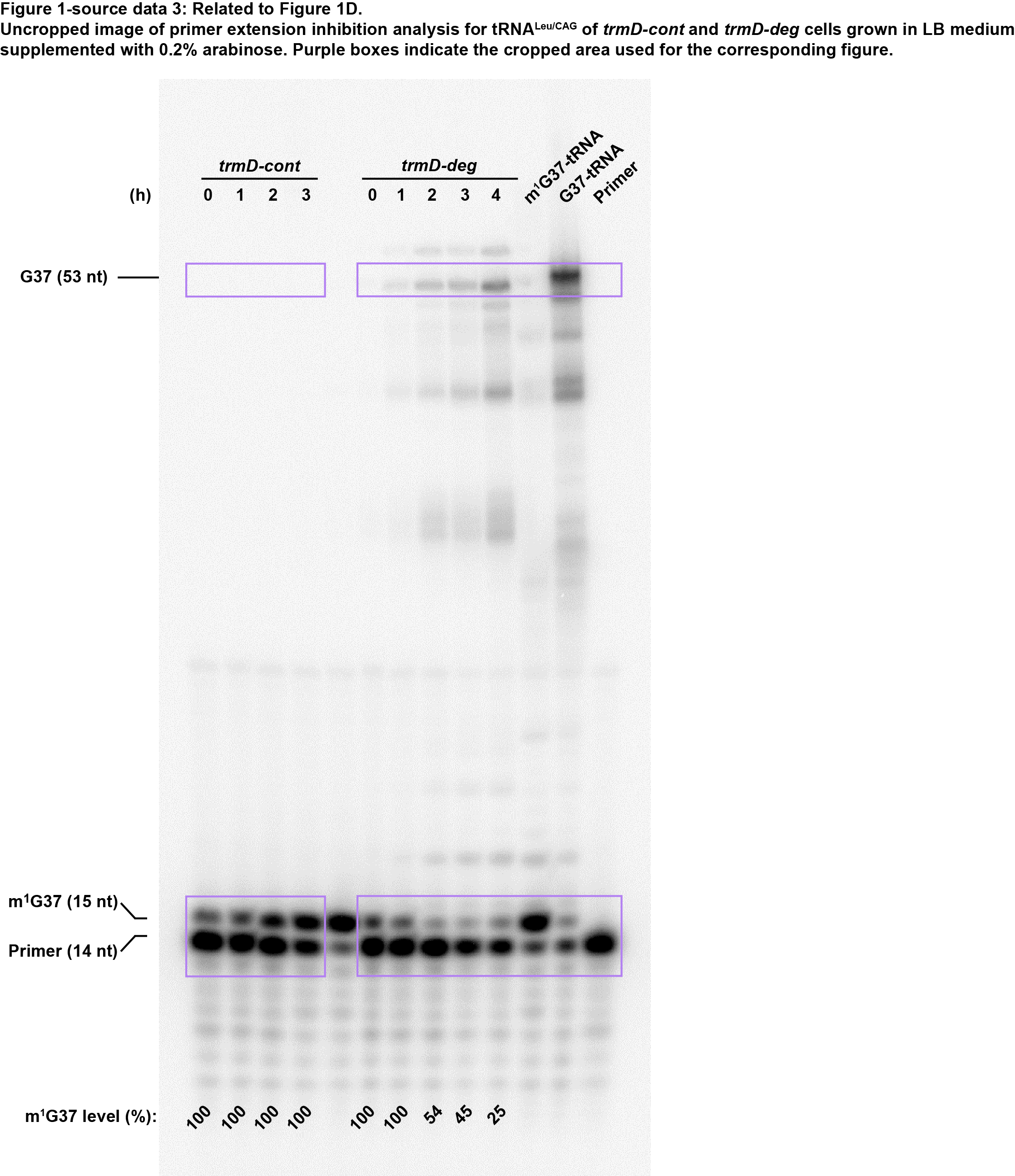

Supplement: Source data 1. [file elife-70619-data1.zip › Figure 1-source data 3.jpg]

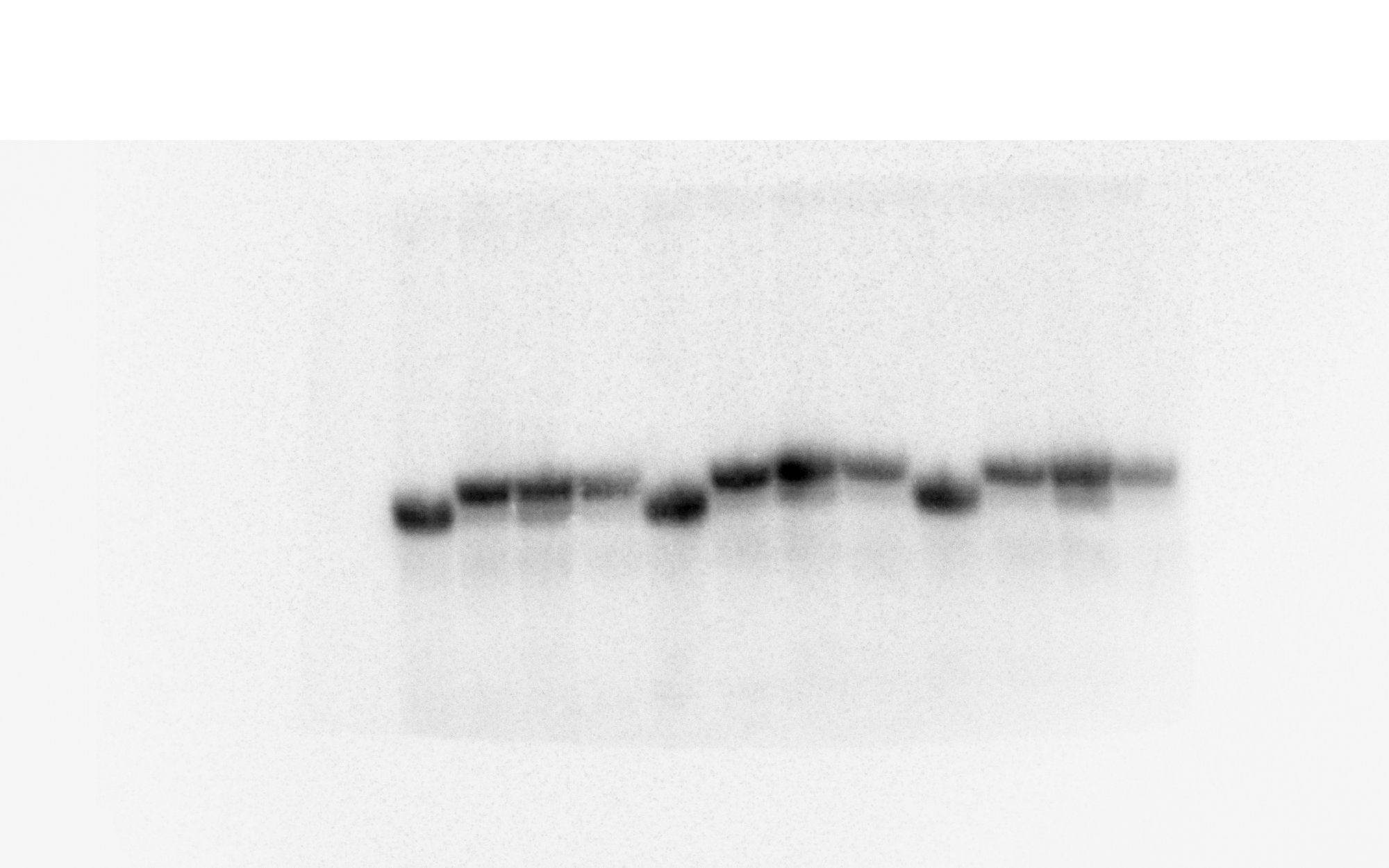

Supplement: Source data 1. [file elife-70619-data1.zip › Figure 4-source data 1 raw.jpg]

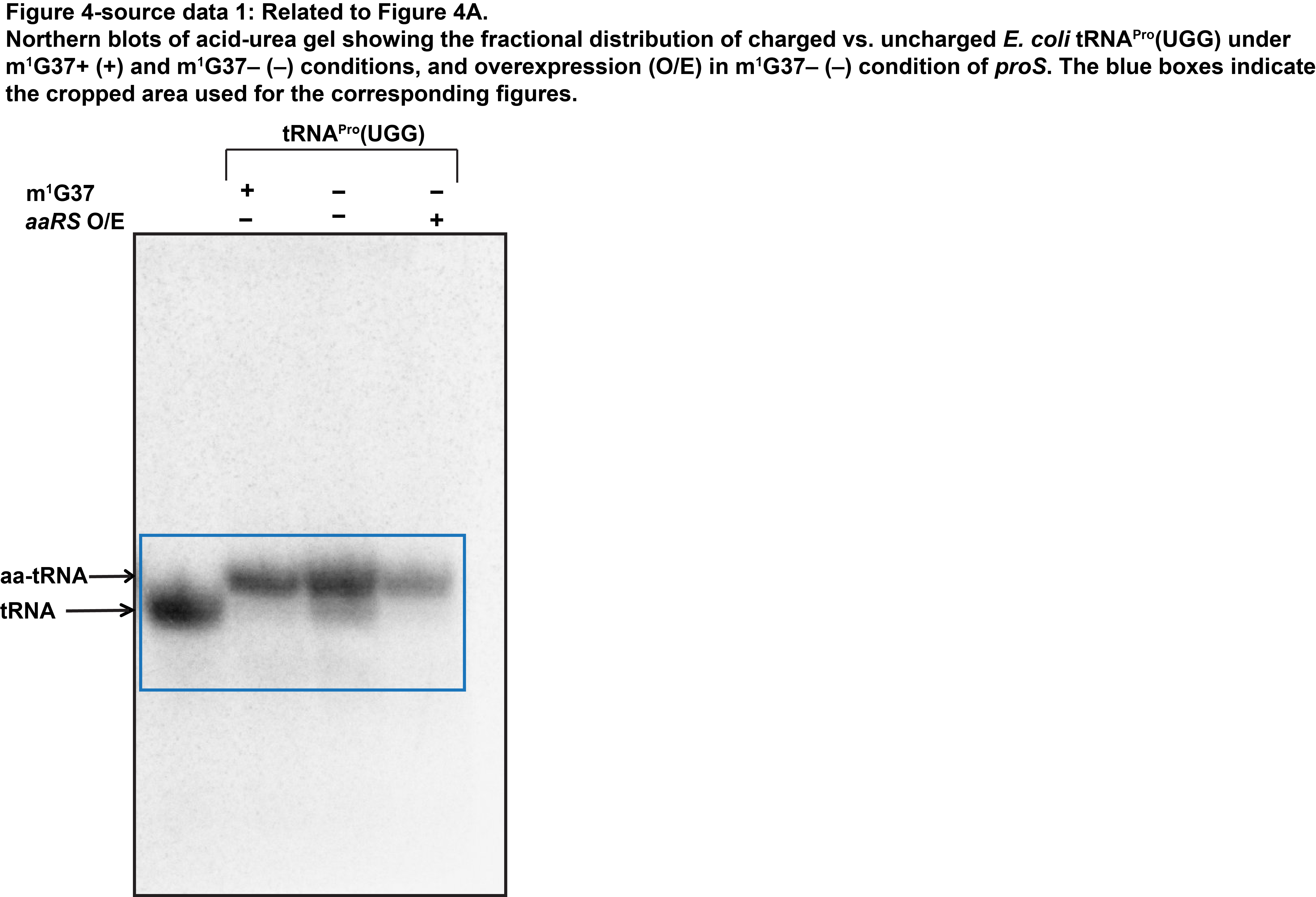

Supplement: Source data 1. [file elife-70619-data1.zip › Figure 4-source data 1.jpg]

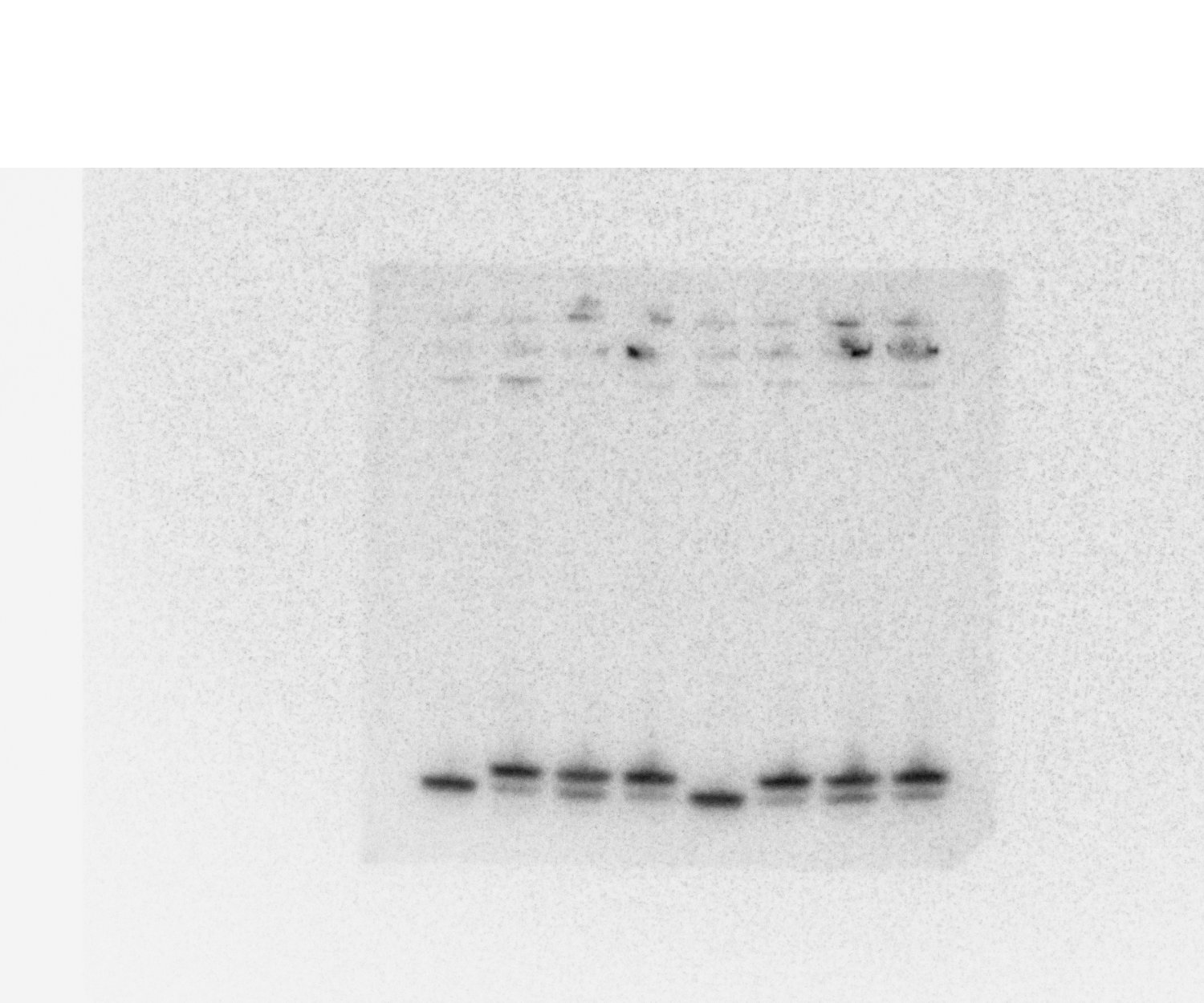

Supplement: Source data 1. [file elife-70619-data1.zip › Figure 4-source data 2 raw.jpg]

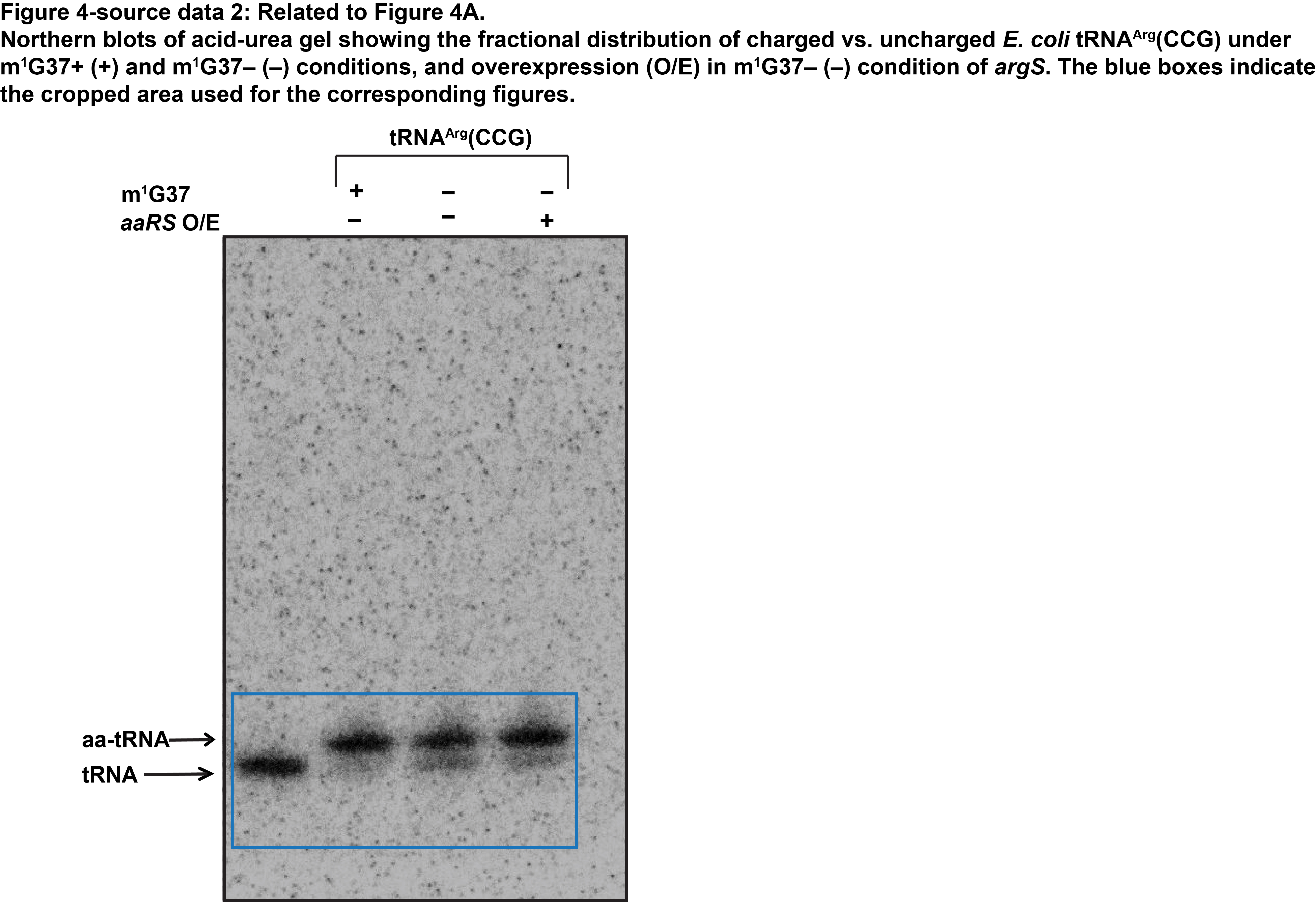

Supplement: Source data 1. [file elife-70619-data1.zip › Figure 4-source data 2.jpg]

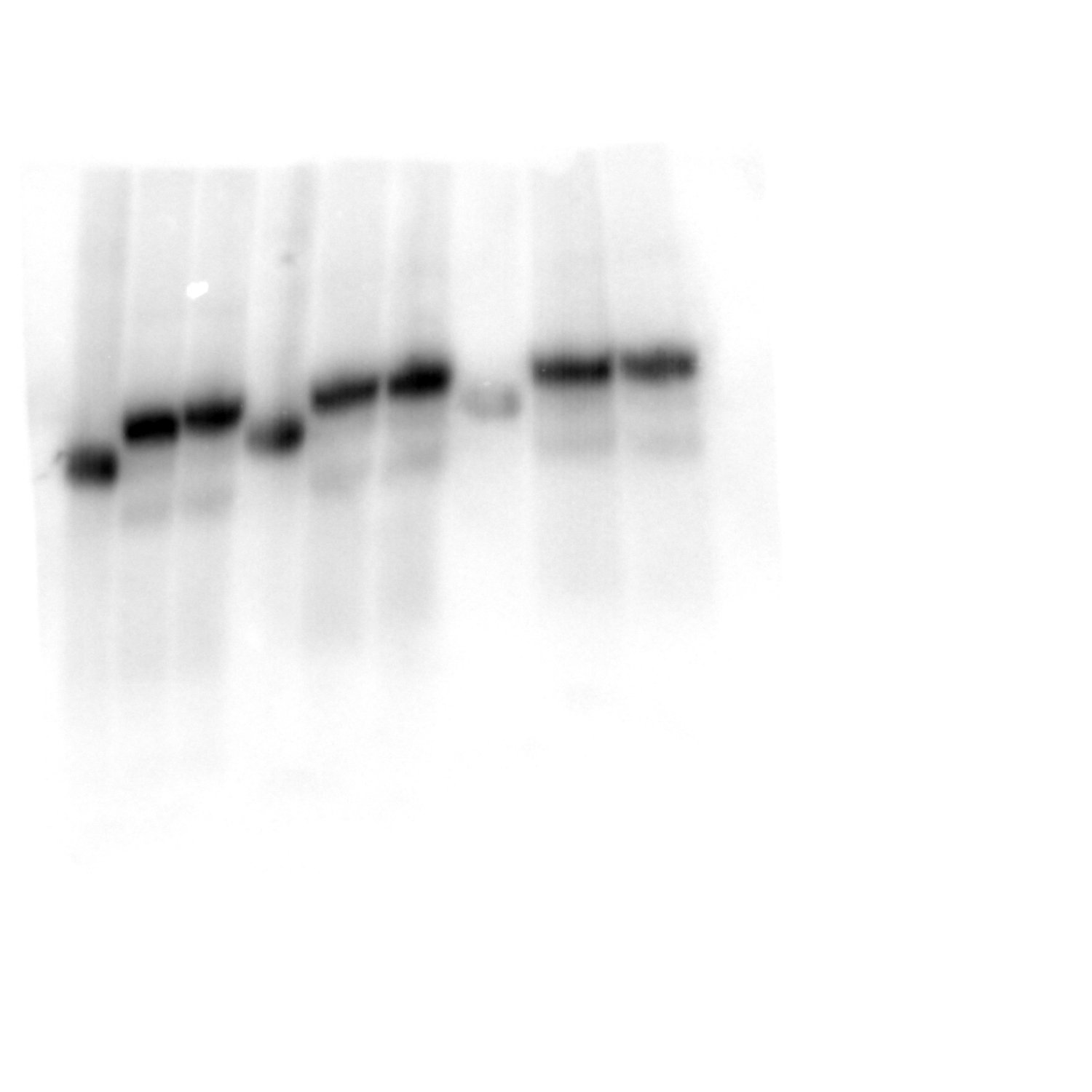

Supplement: Source data 1. [file elife-70619-data1.zip › Figure 4-source data 3 raw.jpg]

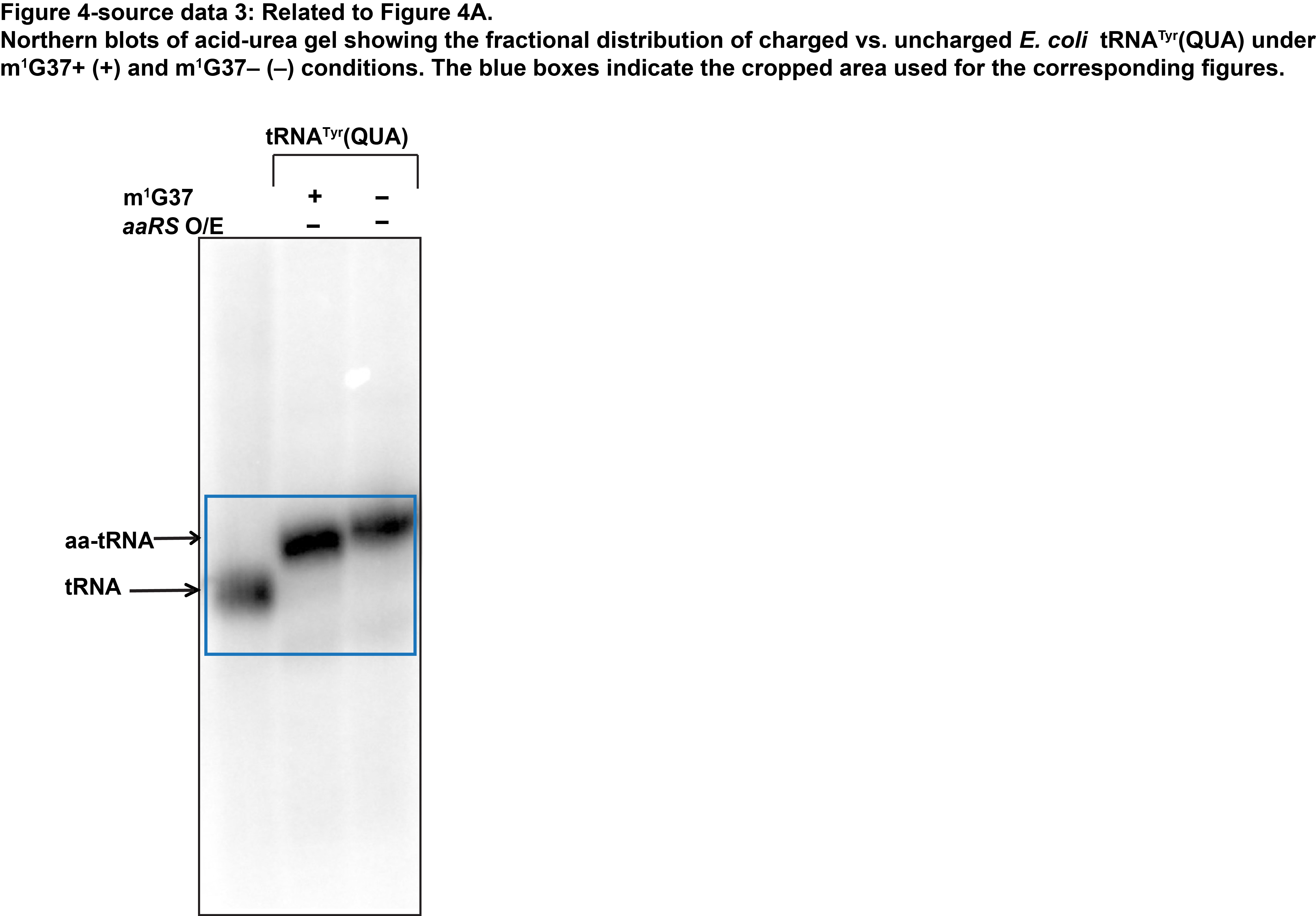

Supplement: Source data 1. [file elife-70619-data1.zip › Figure 4-source data 3.jpg]

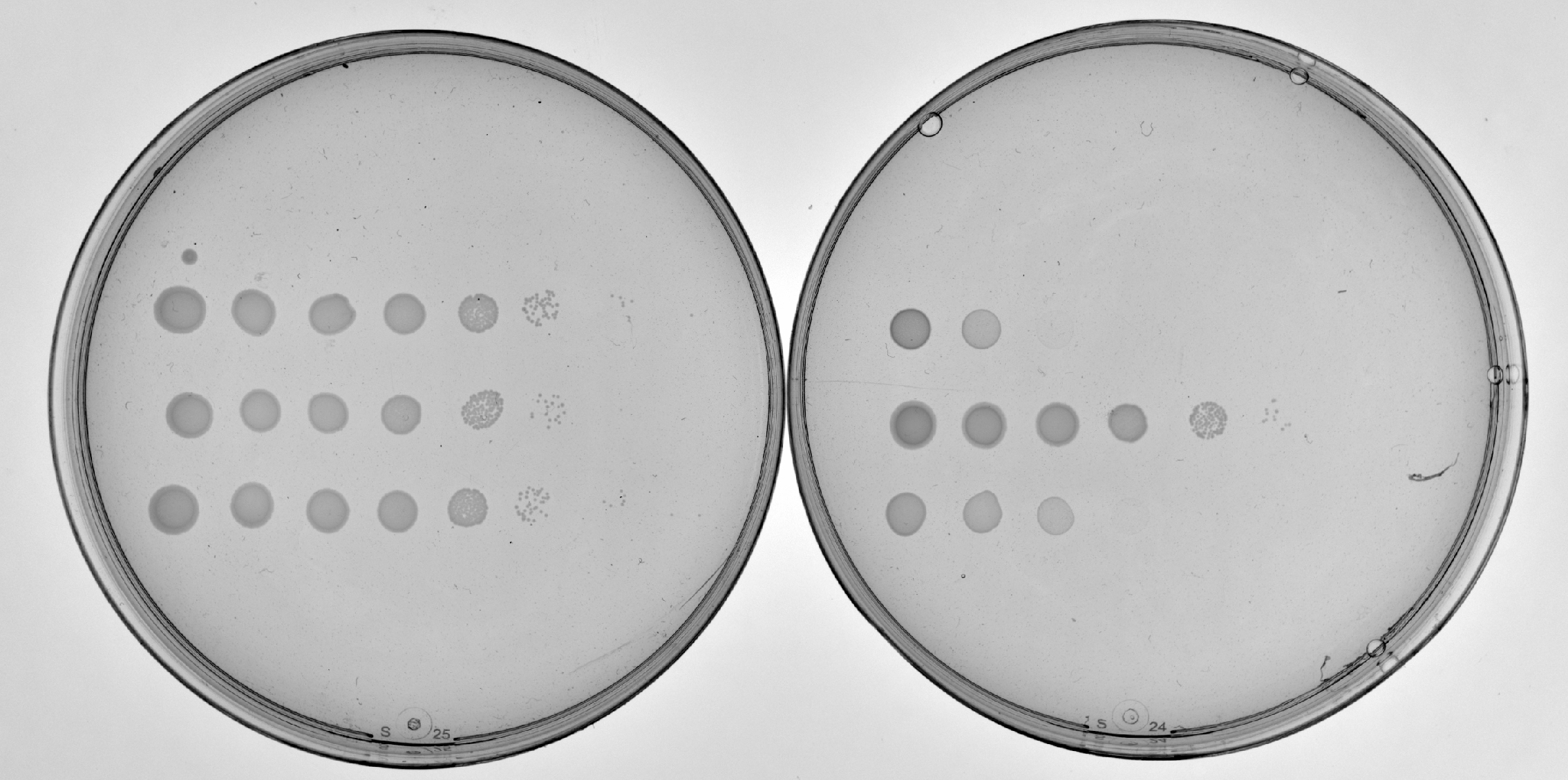

Supplement: Source data 1. [file elife-70619-data1.zip › Figure 4-source data 6 raw.jpg]

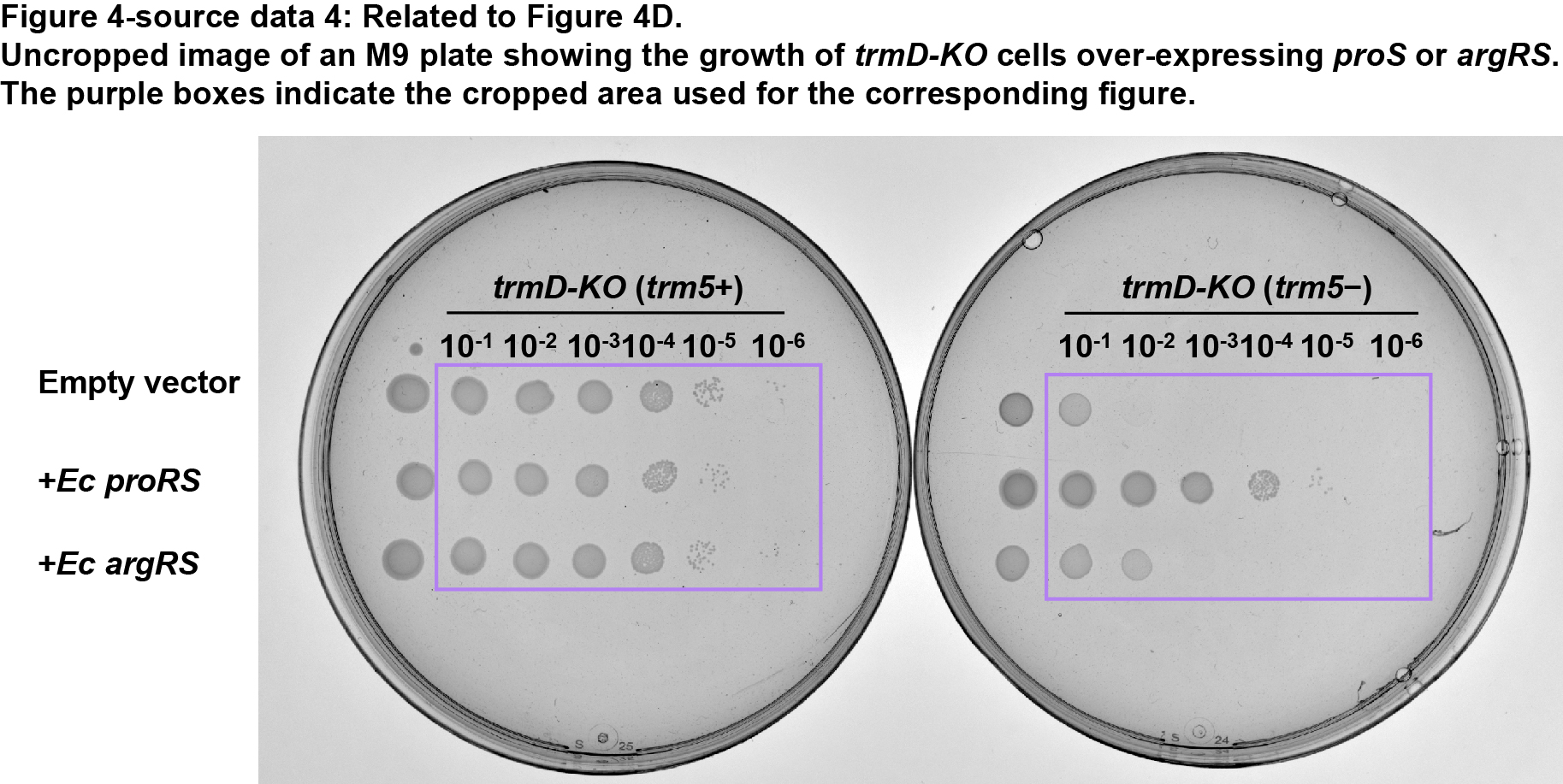

Supplement: Source data 1. [file elife-70619-data1.zip › Figure 4-source data 6.jpg]
