# Supplementary material for: Loss of N1-methylation of G37 in tRNA induces ribosome stalling and reprograms gene expression
Source: eLife. 2021 Aug 12;10:e70619. doi: 10.7554/eLife.70619 (PMC8384417; doi:10.7554/eLife.70619)
Supplement: Transparent reporting form [file elife-70619-transrepform.docx]

***eLife’s* transparent reporting form**

We encourage authors to provide detailed information *within their submission* to facilitate the interpretation and replication of experiments. Authors can upload supporting documentation to indicate the use of appropriate reporting guidelines for health-related research (see [EQUATOR Network](http://www.equator-network.org/%20)), life science research (see the [BioSharing Information Resource](https://biosharing.org/)), or the [ARRIVE guidelines](http://www.plosbiology.org/article/info:doi/10.1371/journal.pbio.1000412) for reporting work involving animal research. Where applicable, authors should refer to any relevant reporting standards documents in this form.

If you have any questions, please consult our Journal Policies and/or contact us: [editorial@elifesciences.org](mailto:editorial@elifesciences.org).

**Sample-size estimation**

- You should state whether an appropriate sample size was computed when the study was being designed
- You should state the statistical method of sample size computation and any required assumptions
- If no explicit power analysis was used, you should describe how you decided what sample (replicate) size (number) to use

Please outline where this information can be found within the submission (e.g., sections or figure legends), or explain why this information doesn’t apply to your submission:

1. Appropriate sample size was provided in Results “Codon-specific ribosome stalling in m1G37 deficiency” and “Changes of gene expression in m1G37 deficiency consistent with the stringent response”.
2. Appropriate sample size was provided in legends to Figures 3, 4, and 5.
3. The statistical method of sample size computation was provided in Results “Changes of gene expression in m1G37 deficiency consistent with the stringent response”.
4. Statistical power was provided in Results “Changes of gene expression in m1G37 deficiency consistent with the stringent response”.

**Replicates**

- You should report how often each experiment was performed
- You should include a definition of biological versus technical replication
- The data obtained should be provided and sufficient information should be provided to indicate the number of independent biological and/or technical replicates
- If you encountered any outliers, you should describe how these were handled
- Criteria for exclusion/inclusion of data should be clearly stated
- High-throughput sequence data should be uploaded before submission, with a private link for reviewers provided (these are available from both GEO and ArrayExpress)

Please outline where this information can be found within the submission (e.g., sections or figure legends), or explain why this information doesn’t apply to your submission:

1. Biochemical experiments were each performed 3 times.
2. A definition of biological replication is provided in the legend to Figure 2A, where it first appeared.
3. The number of independent replicates is provided in legends to Figures, 3-5.
4. There are no outliers in this work.
5. There is no exclusion of data in this work.
6. High-throughput sequencing data are deposited to GEO as in the “Data availability” statement.

**Statistical reporting**

- Statistical analysis methods should be described and justified
- Raw data should be presented in figures whenever informative to do so (typically when N per group is less than 10)
- For each experiment, you should identify the statistical tests used, exact values of N, definitions of center, methods of multiple test correction, and dispersion and precision measures (e.g., mean, median, SD, SEM, confidence intervals; and, for the major substantive results, a measure of effect size (e.g., Pearson's r, Cohen's d)
- Report exact p-values wherever possible alongside the summary statistics and 95% confidence intervals. These should be reported for all key questions and not only when the p-value is less than 0.05.

Please outline where this information can be found within the submission (e.g., sections or figure legends), or explain why this information doesn’t apply to your submission:

1. Statistical analysis methods for transcriptome analysis are defined in Results “Changes of gene expression in m1G37 deficiency consistent with the stringent response”.
2. Raw data are presented in Figure 1-source data 1-5; Figure 3-source data 1-2; Figure 4-source data 1-4; and Figure 5-source data 1-2.
3. The statistical tests for biochemical experiments are shown in legends to Figures 3, 4, and 5.
4. The statistical tests for transcriptome analysis are shown in Results “Changes of gene expression in m1G37 deficiency consistent with the stringent response”.
5. Exact p-values are provided in Results “Changes of gene expression in m1G37 deficiency consistent with the stringent response”.

(For large datasets, or papers with a very large number of statistical tests, you may upload a single table file with tests, Ns, etc., with reference to sections in the manuscript.)

**Group allocation**

- Indicate how samples were allocated into experimental groups (in the case of clinical studies, please specify allocation to treatment method); if randomization was used, please also state if restricted randomization was applied
- Indicate if masking was used during group allocation, data collection and/or data analysis

Please outline where this information can be found within the submission (e.g., sections or figure legends), or explain why this information doesn’t apply to your submission:

Not relevant.

**Additional data files (“source data”)**

- We encourage you to upload relevant additional data files, such as numerical data that are represented as a graph in a figure, or as a summary table
- Where provided, these should be in the most useful format, and they can be uploaded as “Source data” files linked to a main figure or table
- Include model definition files including the full list of parameters used
- Include code used for data analysis (e.g., R, MatLab)
- Avoid stating that data files are “available upon request”

Please indicate the figures or tables for which source data files have been provided:

1. Raw data are presented in Figure 1-source data 1-5; Figure 3-source data 1-2; Figure 4-source data 1-4; and Figure 5-source data 1-2.
2. Source data are uploaded as a zipped file. Each source data file is linked to a main figure.
3. There is no model in this work.
4. The code R is used for data analysis of transcription profiling. No MatLab is used.
